# Supplementary material for: Common bacterial infections and risk of incident cognitive decline or dementia: a systematic review protocol
Source: BMJ Open. 2019 Sep 12;9(9):e030874. doi: 10.1136/bmjopen-2019-030874 (PMC6747671; doi:10.1136/bmjopen-2019-030874)
Supplement: Supplementary data [file bmjopen-2019-030874supp001.pdf]

Appendix 1. Medline (OVID) search strategy

1. Pneumonia/ or pneumonia, bacterial/
2. Pneumonia.ti,ab
3. Lower respiratory tract infection*.ti,ab
4. (LRTI or LRTIS).ti,ab.
5. Exp urinary tract infections/
6. (Urinary adj5 infection*).ti,ab.
7. (UTI or UTIS).ti,ab
8. exp Cystitis/
9. (bacteriuria or pyuria or cystitis or pyelonephritis or cellulitis).ti,ab.
10. exp cellulitis/
11. (Skin and soft tissue infection).mp.
12. Exp sepsis/
13. (septic* or sepsis or septic?emia or systemic inflammatory response syndrome or blood stream infection or py?emia).ti,ab.
14. 1 or 2 or 3 or 4 or 5 or 6 or 7 or 8 or 9 or 10 or 11 or 12 or 13
15. Exp dementia/
16. Exp prion diseases/
17. (huntington* or kluver-bucy or prion disease or Creutzfeldt-jakob or primary progressive aphasia).ti,ab
18. (Dement* or Alzheimer*).ti,ab
19. (Lewy*adj2 bod*).ti,ab.
20. Cognitive dysfunction/
21. (Mild cognitive impairment or MCI).ti,ab
22. ((cognit* or memory or cerebr* or mental*) adj3 (declin* or impair* or los* or deteriorat* or degenerat* or complain* or disturb* or disorder*)).ti,ab.
23. Cognitive function.ti,ab.
24. 15 or 16 or 17 or 18 or 19 or 20 or 21 or 22 or 23
25. cohort studies/ or longitudinal study/ or follow-up study/ or prospective study/ or retrospective study/ or cohort.ti,ab. or longitudinal.ti,ab. or prospective.ti,ab. or retrospective.ti,ab.
26. Case-Control Studies/ or Control Groups/ or Matched-Pair Analysis/ or ((case* adj5 control*) or (case adj3 comparison*) or control group*).ti,ab.
27. Incidence/ or incidence.ti,ab,kw.
28. (hazard ratio or HR or odds ratio or relative risk or RR).ti,ab.
29. 25 or 26 or 27 or 28
30. 14 and 24 and 29
