# Supplementary material for: Common bacterial infections and risk of incident cognitive decline or dementia: a systematic review protocol
Source: BMJ Open. 2019 Sep 12;9(9):e030874. doi: 10.1136/bmjopen-2019-030874 (PMC6747671; doi:10.1136/bmjopen-2019-030874)
Supplement: Author's manuscript [file bmjopen-2019-030874.draft_revisions.pdf]

BMJ Open

BMJ Open is committed to open peer review. As part of this commitment we make the peer review history of every article we publish publicly available.

When an article is published we post the peer reviewers' comments and the authors' responses online. We also post the versions of the paper that were used during peer review. These are the versions that the peer review comments apply to.

The versions of the paper that follow are the versions that were submitted during the peer review process. They are not the versions of record or the final published versions. They should not be cited or distributed as the published version of this manuscript.

BMJ Open is an open access journal and the full, final, typeset and author-corrected version of record of the manuscript is available on our site with no access controls, subscription charges or pay-per-view fees (http://bmjopen.bmj.com).

If you have any questions on BMJ Open's open peer review process please email info.bmjopen@bmj.com

BMJ Open

Common bacterial infections and risk of incident cognitive decline or dementia: a systematic review protocol

Journal:	*BMJ Open*
Manuscript ID	bmjopen-2019-030874
Article Type:	Protocol
Date Submitted by the Author:	04-Apr-2019
Complete List of Authors:	Muzambi, Rutendo; London School of Hygiene and Tropical Medicine, Non-communicable disease epidemiology<br>Bhaskaran, Krishnan; LSHTM, NCDE<br>Brayne, Carol; University of Cambridge, Institute of Public Health<br>Smeeth, Liam; London School of Hygiene and Tropical Medicine, Epidemiology and Population Health<br>Warren-Gash, Charlotte; London School of Hygiene and Tropical Medicine, Non-communicable disease epidemiology
Keywords:	Dementia < NEUROLOGY, INFECTIOUS DISEASES, EPIDEMIOLOGY, Epidemiology < INFECTIOUS DISEASES

SCHOLARONE™
Manuscripts

Common bacterial infections and risk of incident cognitive decline or dementia: a systematic review protocol

Authors: Rutendo Muzambi[1], Krishnan Bhaskaran[1], Carol Brayne[2], Liam Smeeth[1] Charlotte Warren-Gash[1]

Author affiliations:

1. Faculty of Epidemiology and Population Health, London School of Hygiene and tropical, London, WC1E 7HT, UK
2. Cambridge Institute of Public Health, Cambridge University, Cambridge, United Kingdom

Correspondence to:
Rutendo Muzambi. Email: Rutendo.muzambi@lshtm.ac.uk. Tel: 020 7299 4678

Word Count: 1974 excluding title page, abstract, references and tables.

For peer review only - http://bmjopen.bmj.com/site/about/guidelines.xhtml
For peer review only

ABSTRACT

Introduction The global burden of dementia is rising, emphasising the urgent need to develop effective approaches to risk reduction. Recent evidence suggests that common bacterial infections may increase the risk of dementia, however the magnitude and timing of the association as well as the patient groups affected remains unclear. We will review existing evidence of the association between common bacterial infections and incident cognitive decline or dementia.

Methods and analysis We will conduct a comprehensive search of published and grey literature in the following electronic databases; MEDLINE, EMBASE, Global health, PsycINFO, Web of Science, Scopus, Cochrane Library, the Cumulative Index to Nursing and Allied Health Literature (CINAHL), Open Grey and the British Library of electronic theses databases (ETHOS). There will be no restrictions on the date, language or geographical location of the studies. We will include longitudinal studies with a common clinically symptomatic bacterial infection as an exposure and incident cognitive decline or dementia as an outcome. Study selection, data extraction and risk of bias will be performed independently by two researchers. We will assess the risk of bias using the Cochrane collaboration approach. The overall quality of the studies will be assessed using the Grading of Recommendations, Assessment, Development and Evaluations (GRADE) criteria. We will explore the heterogeneity of relevant studies and, if feasible, a meta-analysis will be performed, otherwise we will present a narrative synthesis. We will group the results by exposure and outcome definitions and differences will be described by population subgroups and outcomes.

Ethics and dissemination Ethical approval will not be required as this is a systematic review of existing research in the public domain. Results will be disseminated in a peer-reviewed journal and presented at national and international meetings and conferences.

PROSPERO registration number CRD42018119294

Keywords dementia, Alzheimer’s disease, bacterial infections, systematic review protocol

For peer review only

For peer review only - http://bmjopen.bmj.com/site/about/guidelines.xhtml

ARTICLE SUMMARY

Strengths and limitations

- To the best of our knowledge, this will be the first systematic review assessing the association between common clinically symptomatic bacterial infections and the risk of incident cognitive decline and dementia in longitudinal studies.
- We will be performing a comprehensive search of published and grey literature with no restrictions on date, language or geographical location.
- The review will develop existing evidence to generate better knowledge on the magnitude and direction of the relationship between common bacterial infections and subsequent cognitive decline or dementia.
- Heterogeneity in the way infections, cognitive decline and dementia are defined is expected, which could affect the feasibility of performing a meta-analysis and interpretation of findings.
- There may be difficulty ascertaining whether lower respiratory tract infections are bacterial or viral.

For peer review only

INTRODUCTION

Rationale

Dementia is a clinical syndrome that significantly contributes to disability and dependence worldwide, with a devastating impact on individuals, caregivers and healthcare services.[1-3] It is characterised by a progressive deterioration in cognition and behaviour that interferes with an individual's ability to perform activities of daily living.[4] In 2015, it was estimated that around 47.5 million people were living with dementia, and this number is projected to rise to 135.5 million by 2050, worldwide.[5]

Age is the single biggest risk factor for dementia, with the risk doubling every 5 years after the age of 65.[6] Despite this, there have been some indications that the risk for dementia can be reduced, with more recent cohorts in the UK demonstrating significantly lower risk at any given age.[7] It has become clear from clinical and neuropathological studies that the risk for dementia is complex, not driven by genetics, vascular factors or age alone. Given the rapidly increasing ageing population and the absence of pharmacological treatments that can delay the onset or progression of dementia, identification of effective strategies to reduce risk as age increases has become a public health priority.[8] As a result, recent literature has focused on modifiable risk factors. It has been estimated that around a third of dementia cases worldwide can be attributed to modifiable risk factors.[9-11]

Bacterial infections have been identified as potential risk factors for dementia. For the past few decades, a large body of research has been published on the association between bacterial pathogens and Alzheimer's disease, particularly in post mortem brain tissue.[12-14] Despite this, the temporality of this relationship remains unclear due to the cross-sectional nature of the data collected in these studies. A recent meta-analysis of 27 serological, cerebrospinal fluid and post-mortem brain studies found that chlamydia pneumonia and spirochetal infection were associated with a five-fold (5.66; 95%CI:1.83-17.51) and ten-fold (OR: 10.61; 95% CI 3.38-33.29) increased risk of Alzheimer's disease, respectively.[15] However, temporality could not be assessed in these studies. The meta-analysis was further limited by the inclusion of studies with small sample sizes and the focus on Alzheimer's disease rather than all types of dementia.

Common bacterial infections are well recognised to be associated with acute changes in cognition, manifested as delirium, among older adults.[16] In turn, delirium is strongly associated with an increased risk of subsequent cognitive decline and dementia.[17-19] This raises the challenge of disentangling the relative contribution of the (potentially reversible) ageing immune system's response to acute infections, from ongoing neuropathological processes, both of which may affect cognition.

Longitudinal studies with a follow up time sufficient enough for delirium to resolve, are important in distinguishing between delirium and long-term cognitive impairment. Additionally, longitudinal studies that compare incidence of cognitive decline or dementia in individuals with and without common bacterial infections provide evidence for the temporality and magnitude of this association. Although, the prevalence of common bacterial infections, such as pneumonia and urinary tract infections, in individuals with

For peer review only - http://bmjopen.bmj.com/site/about/guidelines.xhtml

dementia is well known,[20, 21] the incidence of cognitive decline and dementia is less established.

To date, no systematic review has been performed on the incidence of cognitive decline and dementia in individuals with a common bacterial infection causing clinical illness. As these infections frequently occur in the community, early recognition, treatment or prevention of common bacterial infections could have important public health implications in reducing the burden of dementia.

Objectives

The primary objective of the proposed systematic review is to summarise the current literature investigating the association between common clinically symptomatic bacterial infections (sepsis, lower respiratory tract infections, urinary tract infections and skin and soft tissue infections) and incident cognitive decline and dementia in longitudinal studies. A secondary objective will be to identify gaps in literature and recommendations for future research on this topic.

METHODS

The present systematic review protocol was developed according to the Preferred Reporting Items for Systematic Reviews and Meta-analyses Protocols statement and was registered on the PROSPERO database.[22, 23]Any amendments to the protocol will be updated in PROSPERO.

We will report the systematic review in line with the Preferred Reporting Items for Systematic Reviews and Meta-Analyses statement.[24] If a meta-analysis is feasible, it will be reported according to the Meta-analysis of Observational Studies in Epidemiology (MOOSE) statement.[25]

Eligibility criteria

Studies will be eligible for inclusion into the present study if they meet the following inclusion criteria:

Study design
We will include only longitudinal studies (retrospective and prospective cohort studies, randomised controlled trials and case control studies) in which the exposure is ascertained at least 3 months prior to the occurrence of the outcome events in order to investigate the temporal relationship between common bacterial infections and subsequent cognitive decline or dementia.

Study population
We will include human studies of adults aged 18 years and over with no restrictions on the sex, ethnicity, prior health status or residence of participants. We will include studies conducted in any healthcare setting.

For peer review only

For peer review only - http://bmjopen.bmj.com/site/about/guidelines.xhtml

Exposure
We will include studies in which exposure is defined as symptomatic illness due to common bacterial infections, either suspected clinically or confirmed by isolation of a bacterial pathogen. Types of bacterial infections will be subdivided into sepsis, lower respiratory tract infections, skin and soft tissue infections and urinary tract infections. We will exclude studies that focus on a specific bacterial pathogen as the exposure rather than the symptomatic disease (i.e isolation of a bacterium by polymerase chain reaction (PCR) alone in the absence of clinical symptoms).

Comparators
Comparators will include individuals or person time unexposed to common bacterial infections.

Outcomes
We will have two primary outcomes. These will be (1) incident cognitive decline and (2) incident dementia (all types). Cognitive decline will be defined clinically. Dementia will also be defined clinically, with or without neuroimaging or histopathology. If a sufficient number of eligible studies are available, dementia will be subdivided into dementia types.

Literature searches
We will systematically search electronic databases of published and grey literature from inception to March 2019. The following databases will be searched: MEDLINE (Ovid interface), EMBASE (Ovid interface), Global health (Ovid interface), PsycINFO (Ovid Interface), Web of Science, Scopus, Cochrane Library, the Cumulative Index to Nursing and Allied Health Literature (CINAHL), Open Grey and the British Library of Electronic Theses databases (ETHOS). Additionally, we will search the reference lists of the included studies to identify any relevant articles not captured in the search strategy. We will search the databases using subject headings, where possible, and keywords related to the exposure, outcome and study design. These search terms will be combined using Boolean logical operators.

We developed a search strategy for the MEDLINE database which is provided in appendix 1. We will translate our search strategy in all databases using search syntaxes specific to each database. No restrictions will be placed on the geographical location, language or date of publication of the studies. Any potentially relevant non-English studies will be translated.

Study records

Data management
We will import the search results into the reference manager software EndNote (X8.0.2). Duplicate entries will be identified and removed.

Study selection
Two researchers will independently screen all titles and abstracts against the eligibility criteria. The researchers will then independently screen the full text articles of potentially eligible articles and decide on whether the inclusion criteria has been met. Any disagreements between the reviewers will be discussed and if necessary a third reviewer

For peer review only - http://bmjopen.bmj.com/site/about/guidelines.xhtml
For peer review only

will be consulted. Reasons for exclusion of studies will be recorded. We will document the study selection process using the Preferred Reporting Items for Systematic Reviews and Meta-Analyses flow diagram.[24] If multiple papers arise from the same population, we will include the paper with the largest sample size and most detailed information about the exposure and outcome.

Data collection process

Two researchers will independently extract data from included papers onto a predesigned form. If necessary, authors will be contacted directly to obtain any missing information.

Data items

We will use the Population, Exposure, Comparator, Outcomes and Study characteristics (PECOS) framework to design our data extraction form. The following information will be extracted:

1. Population: age (mean, median or range), sex, inclusion and exclusion criteria.
2. Exposure: definition of exposure, type of bacterial infection, number of exposed.
3. Comparators: identification and definition of comparator, number of comparators.
4. Outcomes: definition of outcome and identification of cognitive decline and dementia, number of participants with the outcome.
5. Study Characteristics: authors, name of study, year of publication, study design, type of longitudinal study, healthcare setting, country, sample size, duration of follow up.

Regarding the study results, we will extract unadjusted and adjusted estimates and their corresponding 95% CI for each exposure and outcome. Additionally, we will extract data on the confounding variables adjusted for in each study as well as data on sub-group or sensitivity analyses.

Risk of bias in individual studies

Two researchers will independently assess the risk of bias in line with the Cochrane Collaboration approach.[26, 27] We will examine the risk of bias for randomised controlled trials using the following domains: sequence generation; allocation concealment; blinding of participants and personnel; blinding of outcome assessment; incomplete outcome data; selective outcome reporting and other potential threats to validity.[27] We will assess the risk of bias for observational studies using the following domains: confounding, selection of participants, misclassification of variables, missing data, reverse causation, generalisability and study power.

Data synthesis and meta-bias(es)

For peer review only

For peer review only - http://bmjopen.bmj.com/site/about/guidelines.xhtml

We will group studies according to the outcome (cognitive decline or dementia) and exposure (type of common bacterial infection) and synthesise them narratively. Data will be summarised in pre-defined tables. We will consider subgroup analyses according to age-group, gender, dementia subtype and risk of bias.

A meta-analysis will be considered feasible if there are sufficient studies that are homogeneous in terms of study design, type of common bacterial infection and type of outcome. We will pool effect measures (odds ratios, risk ratios or hazard ratios) from the studies in order to perform the meta-analysis.

We will assess statistical heterogeneity through the use of forests plots, chi-squared tests and I^2 statistic. Depending on the level of heterogeneity, a fixed or random effects model will be selected to calculate the pooled incidence and corresponding 95% CI. A chi square test with a p value of 0.1 will be considered statistically significant. We will consider an I^2 value of >25% to indicate moderate heterogeneity, which will guide the use of a random effects model.[28, 29] We will assess publication bias and small study effects using funnel plots, provided that there are 10 or more studies eligible for inclusion into the meta-analysis.[30]

Confidence in cumulative evidence

We will assess the overall quality of evidence for each outcome using the Grading of Recommendations, Assessment, Development and Evaluation (GRADE) tool.[31] The domains assessed will include study limitations, inconsistency, indirectness, imprecision and publication bias.[32] The strength of the evidence will be categorised as high, moderate or low.

ETHICS AND DISSEMINATION

This systematic review will provide evidence for the role of common bacterial infections in the development of cognitive decline and dementia. The systematic review will be submitted for publication in a peer-reviewed journal and the results may be presented at national and international conferences and meetings relevant to the field. This review will highlight gaps in current literature and identify future research directions. Ethical approval will not be required as the review will summarise data from previous studies.

Author contribution

CWG, KB and LS, conceived and designed the study, revised the protocol and search strategy critically. RM conceived and designed the study, drafted and revised the protocol, and developed and revised the search strategy. CB contributed to the design of the study and revised the protocol and search strategy critically. The final version of the protocol was read and approved by all authors.

Funding

For peer review only - http://bmjopen.bmj.com/site/about/guidelines.xhtml

Rutendo Muzambi is supported by an Alzheimer's Society PhD studentship 379 (AS-PhD-17-013). The funder played no role in developing the protocol.

Competing interests

No conflict of interest was reported by any of the authors.

For peer review only

References

1. Sousa RM, Ferri CP, Acosta D, et al. Contribution of chronic diseases to disability in elderly people in countries with low and middle incomes: a 10/66 Dementia Research Group population-based survey. *Lancet* 2009;374:1821-30.

2. Harwood RH, Sayer AA, Hirschfeld M. Current and future worldwide prevalence of dependency, its relationship to total population, and dependency ratios. *Bull World Health Organ* 2004;82:251-8.

3. Global, regional, and national burden of Alzheimer's disease and other dementias, 1990-2016: a systematic analysis for the Global Burden of Disease Study 2016. *Lancet Neurol* 2019;18:88-106.

4. World Health Organization. Global action plan on the public health response to dementia 2017-2025. Geneva: World Health Organization; 2017.

5. Prince M, Guerchet M, Prina M. Policy Brief for Heads of Government: The Global Impact of Dementia 2013–2050. London: Alzheimer's Disease International 2013. http://www.alz.co.uk/research/G8-policy-brief (accessed 25 Jan 2019).

6. Hugo J, Ganguli M. Dementia and cognitive impairment: epidemiology, diagnosis, and treatment. *Clin Geriatr Med* 2014;30:421-42.

7. Matthews FE, Stephan BC, Robinson L, et al. A two decade dementia incidence comparison from the Cognitive Function and Ageing Studies I and II. *Nat Commun* 2016;7:11398.

8. World Health Organization. Dementia: a public health priority 2012. http://www.who.int/mental_health/publications/dementia_report_2012/en/ (accessed 6 Feb 2019).

9. Barnes DE, Yaffe K. The projected effect of risk factor reduction on Alzheimer's disease prevalence. *Lancet Neurol* 2011;10:819-28.

10. Norton S, Matthews FE, Barnes DE, et al. Potential for primary prevention of Alzheimer's disease: an analysis of population-based data. *Lancet Neurol* 2014;13:788-94.

11. Livingston G, Sommerlad A, Orgeta V, et al. Dementia prevention, intervention, and care. *Lancet* 2017;390:2673-734.

12. Sochocka M, Zwolinska K, Leszek J. The Infectious Etiology of Alzheimer's Disease. *Curr Neuropharmacol* 2017;15:996-1009.

13. Lim SL, Rodriguez-Ortiz CJ, Kitazawa M. Infection, systemic inflammation, and Alzheimer's disease. *Microbes Infect* 2015;17:549-56.

For peer review only - http://bmjopen.bmj.com/site/about/guidelines.xhtml

14. Mawanda F, Wallace R. Can Infections Cause Alzheimer's Disease? *Epidemiol Rev* 2013;35:161-80.

15. Maheshwari P, Eslick GD. Bacterial infection and Alzheimer's disease: a meta-analysis. *J Alzheimers Dis* 2015;43:957-66.

16. van Gool WA, van de Beek D, Eikelenboom P. Systemic infection and delirium: when cytokines and acetylcholine collide. *Lancet* 2010;375:773-5.

17. Girard TD, Jackson JC, Pandharipande PP, et al. Delirium as a predictor of long-term cognitive impairment in survivors of critical illness. *Crit Care Med* 2010;38:1513-20.

18. MacLullich AM, Beaglehole A, Hall RJ, et al. Delirium and long-term cognitive impairment. *Int Rev Psychiatry* 2009;21:30-42.

19. Davis DH, Muniz Terrera G, Keage H, et al. Delirium is a strong risk factor for dementia in the oldest-old: a population-based cohort study. *Brain* 2012;135:2809-16.

20. Foley NC, Affoo RH, Martin RE. A Systematic Review and Meta-Analysis Examining Pneumonia-Associated Mortality in Dementia. *Dement Geriatr Cogn Disord* 2015;39:52-67.

21. Chae JH, Miller BJ. Beyond Urinary Tract Infections (UTIs) and Delirium: A Systematic Review of UTIs and Neuropsychiatric Disorders. *J Psychiatr Pract* 2015;21:402-11.

22. Moher D, Shamseer L, Clarke M, et al. Preferred reporting items for systematic review and meta-analysis protocols (PRISMA-P) 2015 statement. *Syst Rev* 2015;4:1.

23. Booth A, Clarke M, Dooley G, et al. The nuts and bolts of PROSPERO: an international prospective register of systematic reviews. *Syst Rev* 2012;1:2.

24. Moher D, Liberati A, Tetzlaff J, et al. Preferred reporting items for systematic reviews and meta-analyses: the PRISMA statement. *PLoS Med* 2009;6:e10000097.

25. Stroup DF, Berlin JA, Morton SC, et al. Meta-analysis of observational studies in epidemiology: a proposal for reporting. Meta-analysis Of Observational Studies in Epidemiology (MOOSE) group. *Jama* 2000;283:2008-12.

26. Sterne JA, Hernán MA, Reeves BC, et al. ROBINS-I: a tool for assessing risk of bias in non-randomised studies of interventions. *BMJ* 2016;355.

27. Higgins JPT, Altman DG, Gøtzsche PC, et al. The Cochrane Collaboration's tool for assessing risk of bias in randomised trials. *BMJ* 2011;343.

28. Riley RD, Higgins JPT, Deeks JJ. Interpretation of random effects meta-analyses. *BMJ* 2011;342.

For peer review only

29. Higgins J, Green S. Section 9.5.2. Identifying and measuring heterogeneity in Cochrane handbook for systematic reviews of interventions 2011. https://handbook-5-1.cochrane.org/chapter_9/9_5_2_identifying_and_measuring_heterogeneity.htm; (accessed 8 Mar 2019).

30. Sterne JA, Egger M. Funnel plots for detecting bias in meta-analysis: guidelines on choice of axis. *J Clin Epidemiol* 2001;54:1046-55.

31. Guyatt GH, Oxman AD, Vist GE, et al. GRADE: an emerging consensus on rating quality of evidence and strength of recommendations. *BMJ* 2008;336:924-6.

32. Balshem H, Helfand M, Schünemann HJ, et al. GRADE guidelines: 3. Rating the quality of evidence. *J Clin Epidemiol* 2011;64:401-6.

For peer review only

Appendix 1. Medline (OVID) search strategy

1. Pneumonia/ or pneumonia, bacterial/
2. Pneumonia.ti,ab
3. Lower respiratory tract infection*.ti,ab
4. (LRTI or LRTIS).ti,ab.
5. Exp urinary tract infections/
6. (Urinary adj5 infection*).ti,ab.
7. (UTI or UTIS).ti,ab
8. exp Cystitis/
9. (bacteriuria or pyuria or cystitis or pyelonephritis or cellulitis).ti,ab.
10. exp cellulitis/
11. (Skin and soft tissue infection).mp.
12. Exp sepsis/
13. (septic* or sepsis or septic?emia or systemic inflammatory response syndrome or blood stream infection or py?emia).ti,ab.
14. 1 or 2 or 3 or 4 or 5 or 6 or 7 or 8 or 9 or 10 or 11 or 12 or 13
15. Exp dementia/
16. Exp prion diseases/
17. (huntington* or kluver-bucy or prion disease or Creutzfeldt-jakob or primary progressive aphasia).ti,ab
18. (Dement* or Alzheimer*).ti,ab
19. (Lewy*adj2 bod*).ti,ab.
20. Cognitive dysfunction/
21. (Mild cognitive impairment or MCI).ti,ab
22. ((cognit* or memory or cerebr* or mental*) adj3 (declin* or impair* or los* or deteriorat* or degenerat* or complain* or disturb* or disorder*)).ti,ab.
23. Cognitive function.ti,ab.
24. 15 or 16 or 17 or 18 or 19 or 20 or 21 or 22 or 23
25. cohort studies/ or longitudinal study/ or follow-up study/ or prospective study/ or retrospective study/ or cohort.ti,ab. or longitudinal.ti,ab. or prospective.ti,ab. or retrospective.ti,ab.
26. Case-Control Studies/ or Control Groups/ or Matched-Pair Analysis/ or ((case* adj5 control*) or (case adj3 comparison*) or control group*).ti,ab.
27. Incidence/ or incidence.ti,ab,kw.
28. (hazard ratio or HR or odds ratio or relative risk or RR).ti,ab.
29. 25 or 26 or 27 or 28
30. 14 and 24 and 29

For peer review only

PRISMA-P (Preferred Reporting Items for Systematic review and Meta-Analysis Protocols) 2015 checklist: recommended items to address in a systematic review protocol*

Section and topic	Item No	Checklist item		
**ADMINISTRATIVE INFORMATION**				
Title:				
Identification	1a	Identify the report as a protocol of a systematic review	✓	P1
Update	1b	If the protocol is for an update of a previous systematic review, identify as such	-	-
Registration	2	If registered, provide the name of the registry (such as PROSPERO) and registration number	✓	P2
Authors:				
Contact	3a	Provide name, institutional affiliation, e-mail address of all protocol authors; provide physical mailing address of corresponding author	✓	P1
Contributions	3b	Describe contributions of protocol authors and identify the guarantor of the review	✓	P8
Amendments	4	If the protocol represents an amendment of a previously completed or published protocol, identify as such and list changes; otherwise, state plan for documenting important protocol amendments	-	-
Support:				
Sources	5a	Indicate sources of financial or other support for the review	✓	P9
Sponsor	5b	Provide name for the review funder and/or sponsor	✓	P9
Role of sponsor or funder	5c	Describe roles of funder(s), sponsor(s), and/or institution(s), if any, in developing the protocol	✓	P9
**INTRODUCTION**				
Rationale	6	Describe the rationale for the review in the context of what is already known	✓	P4/5
Objectives	7	Provide an explicit statement of the question(s) the review will address with reference to participants, interventions, comparators, and outcomes (PICO)	✓	P5
**METHODS**				
Eligibility criteria	8	Specify the study characteristics (such as PICO, study design, setting, time frame) and report characteristics (such as years considered, language, publication status) to be used as criteria for eligibility for the review	✓	P5/6
Information sources	9	Describe all intended information sources (such as electronic databases, contact with study authors, trial registers or other grey literature sources) with planned dates of coverage	✓	P6

For peer review only

Search strategy	10	Present draft of search strategy to be used for at least one electronic database, including planned limits, such that it could be repeated	✓	P6 (and in appendices)
Study records:				
Data management	11a	Describe the mechanism(s) that will be used to manage records and data throughout the review	✓	P6
Selection process	11b	State the process that will be used for selecting studies (such as two independent reviewers) through each phase of the review (that is, screening, eligibility and inclusion in meta-analysis)	✓	P6/7
Data collection process	11c	Describe planned method of extracting data from reports (such as piloting forms, done independently, in duplicate), any processes for obtaining and confirming data from investigators	✓	P7
Data items	12	List and define all variables for which data will be sought (such as PICO items, funding sources), any pre-planned data assumptions and simplifications	✓	P7
Outcomes and prioritization	13	List and define all outcomes for which data will be sought, including prioritization of main and additional outcomes, with rationale	✓	P6
Risk of bias in individual studies	14	Describe anticipated methods for assessing risk of bias of individual studies, including whether this will be done at the outcome or study level, or both; state how this information will be used in data synthesis	✓	P7
Data synthesis	15a	Describe criteria under which study data will be quantitatively synthesised	✓	P8
	15b	If data are appropriate for quantitative synthesis, describe planned summary measures, methods of handling data and methods of combining data from studies, including any planned exploration of consistency (such as I^2, Kendall's τ)	✓	P8
	15c	Describe any proposed additional analyses (such as sensitivity or subgroup analyses, meta-regression)	✓	P8
	15d	If quantitative synthesis is not appropriate, describe the type of summary planned	✓	P8
Meta-bias(es)	16	Specify any planned assessment of meta-bias(es) (such as publication bias across studies, selective reporting within studies)	✓	P8
Confidence in cumulative evidence	17	Describe how the strength of the body of evidence will be assessed (such as GRADE)	✓	P8

*** It is strongly recommended that this checklist be read in conjunction with the PRISMA-P Explanation and Elaboration (cite when available) for important clarification on the items. Amendments to a review protocol should be tracked and dated. The copyright for PRISMA-P (including checklist) is held by the PRISMA-P Group and is distributed under a Creative Commons Attribution Licence 4.0.**

From: Shamseer L, Moher D, Clarke M, Ghersi D, Liberati A, Petticrew M, Shekelle P, Stewart L, PRISMA-P Group. Preferred reporting items for systematic review and meta-analysis protocols [22] 2015: elaboration and explanation. BMJ. 2015 Jan 2;349(jan02 1):g7647.

For peer review only

For peer review only

BMJ Open

Common bacterial infections and risk of incident cognitive decline or dementia: a systematic review protocol

Journal:	*BMJ Open*
Manuscript ID	bmjopen-2019-030874.R1
Article Type:	Protocol
Date Submitted by the Author:	10-Jun-2019

Complete List of Authors:	Muzambi, Rutendo; London School of Hygiene and Tropical Medicine, Non-communicable disease epidemiology<br>Bhaskaran, Krishnan; LSHTM, NCDE<br>Brayne, Carol; University of Cambridge, Institute of Public Health<br>Smeeth, Liam; London School of Hygiene and Tropical Medicine, Epidemiology and Population Health<br>Warren-Gash, Charlotte; London School of Hygiene and Tropical Medicine, Non-communicable disease epidemiology

**Primary Subject Heading**:	Epidemiology
Secondary Subject Heading:	Neurology, Infectious diseases
Keywords:	Dementia < NEUROLOGY, INFECTIOUS DISEASES, EPIDEMIOLOGY, Epidemiology < INFECTIOUS DISEASES

SCHOLARONE™ Manuscripts

Common bacterial infections and risk of incident cognitive decline or dementia: a systematic review protocol

Authors: Rutendo Muzambi[1], Krishnan Bhaskaran[1], Carol Brayne[2], Liam Smeeth[1] Charlotte Warren-Gash[1]

Author affiliations:

1. Faculty of Epidemiology and Population Health, London School of Hygiene and tropical, London, WC1E 7HT, UK
2. Cambridge Institute of Public Health, Cambridge University, Cambridge, United Kingdom

Correspondence to:
Rutendo Muzambi. Email: Rutendo.muzambi@lshtm.ac.uk. Tel: 020 7299 4678

Word Count: 2570 excluding title page, abstract, references and tables.

For peer review only

ABSTRACT

Introduction The global burden of dementia is rising, emphasising the urgent need to develop effective approaches to risk reduction. Recent evidence suggests that common bacterial infections may increase the risk of dementia, however the magnitude and timing of the association as well as the patient groups affected remains unclear. We will review existing evidence of the association between common bacterial infections and incident cognitive decline or dementia.

Methods and analysis We will conduct a comprehensive search of published and grey literature from inception to 18 March 2019. The following electronic databases will be searched; MEDLINE, EMBASE, Global health, PsycINFO, Web of Science, Scopus, Cochrane Library, the Cumulative Index to Nursing and Allied Health Literature (CINAHL), Open Grey and the British Library of electronic theses databases (ETHOS). There will be no restrictions on the date, language or geographical location of the studies. We will include longitudinal studies with a common clinically symptomatic bacterial infection as an exposure and incident cognitive decline or dementia as an outcome. Study selection, data extraction and risk of bias will be performed independently by two researchers. We will assess the risk of bias using the Cochrane collaboration approach. The overall quality of the studies will be assessed using the Grading of Recommendations, Assessment, Development and Evaluations criteria. We will explore the heterogeneity of relevant studies and, if feasible, a meta-analysis will be performed, otherwise we will present a narrative synthesis. We will group the results by exposure and outcome definitions and differences will be described by subgroups and outcomes.

Ethics and dissemination Ethical approval will not be required as this is a systematic review of existing research in the public domain. Results will be disseminated in a peer-reviewed journal and presented at national and international meetings and conferences.

PROSPERO registration number CRD42018119294

Keywords dementia, Alzheimer’s disease, bacterial infections, systematic review protocol

For peer review only

ARTICLE SUMMARY

Strengths and limitations

- To the best of our knowledge, this will be the first systematic review assessing the association between common clinically symptomatic bacterial infections and the risk of incident cognitive decline and dementia in longitudinal studies.
- We will perform a comprehensive search of published and grey literature with no restrictions on date, language or geographical location.
- The review will develop existing evidence to generate better knowledge on the magnitude and direction of the relationship between common bacterial infections and subsequent cognitive decline or dementia.
- Heterogeneity in the way infections, cognitive decline and dementia are defined is expected, which could affect the feasibility of performing a meta-analysis and interpretation of findings.
- There may be difficulty ascertaining whether lower respiratory tract infections are bacterial or viral.

For peer review only

INTRODUCTION

Rationale

Dementia is a clinical syndrome that significantly contributes to disability and dependence worldwide, with a devastating impact on individuals, caregivers and healthcare services.[1-3] It is characterised by a progressive deterioration in cognition and behaviour that interferes with an individual's ability to perform activities of daily living.[4] In 2015, it was estimated that around 47.5 million people were living with dementia, and this number is projected to rise to 135.5 million by 2050, worldwide.[5]

Age is the single biggest risk factor for dementia, with the risk doubling every 5 years after the age of 65.[6] Despite this, there have been some indications that the risk for dementia can be reduced, with more recent cohorts in the UK demonstrating significantly lower risk at any given age.[7] It has become clear from clinical and neuropathological studies that the risk for dementia is complex, not driven by genetics, vascular factors or age alone. Given the rapidly increasing ageing population and the absence of pharmacological treatments that can delay the onset or progression of dementia, identification of effective strategies to reduce risk as age increases has become a public health priority.[8] As a result, recent literature has focused on modifiable risk factors. It has been estimated that around a third of dementia cases worldwide can be attributed to modifiable risk factors.[9-11]

Bacterial infections have been identified as potential risk factors for dementia. For the past few decades, a large body of research has been published on the association between bacterial pathogens and Alzheimer's disease, particularly in post mortem brain tissue.[12-14] Despite this, the temporality of this relationship remains unclear due to the cross-sectional nature of the data collected in these studies. A recent meta-analysis of 27 serological, cerebrospinal fluid and post-mortem brain studies found that chlamydia pneumonia and spirochaetal infection were associated with a five-fold (OR 5.66; 95%CI:1.83-17.51) and ten-fold (OR 10.61, 95% CI: 3.38-33.29) increased risk of Alzheimer's disease, respectively.[15] However, temporality could not be assessed in these studies. The meta-analysis was further limited by the inclusion of studies with small sample sizes and the focus on Alzheimer's disease rather than all types of dementia.

Common bacterial infections are well recognised to be associated with acute changes in cognition, manifested as delirium, among older adults.[16] In turn, delirium is strongly associated with an increased risk of subsequent cognitive decline and dementia.[17-19] This raises the challenge of disentangling the relative contribution of the (potentially reversible) ageing immune system's response to acute infections, from ongoing neuropathological processes, both of which may affect cognition.

Longitudinal studies with a follow up time sufficient enough for delirium to resolve, are important in distinguishing between delirium and long-term cognitive impairment. Additionally, longitudinal studies that compare incidence of cognitive decline or dementia in individuals with and without common bacterial infections provide evidence for the temporality and magnitude of this association. Although, the prevalence of common bacterial infections, such as pneumonia and urinary tract infections, in individuals with

For peer review only - http://bmjopen.bmj.com/site/about/guidelines.xhtml

dementia is well known,[20, 21] the incidence of cognitive decline and dementia is less established.

The mechanisms by which bacterial infections may increase the risk of cognitive decline and dementia are unclear but may involve inflammation.[22, 23] Bacterial infections may trigger an inflammatory response in the brain resulting in the release of pro-inflammatory mediators and activation of cytotoxic microglia. This may result in deterioration of cognitive function, possibly increasing the risk of dementia.[12]

To date, no systematic review has been performed on the incidence of cognitive decline and dementia in individuals with a common bacterial infection causing clinical illness. As these infections frequently occur in the community, early recognition, treatment or prevention of common bacterial infections could have important public health implications in reducing the burden of dementia.

Objectives

The primary objective of the proposed systematic review is to summarise the current literature investigating the association between common clinically symptomatic bacterial infections (sepsis, lower respiratory tract infections, urinary tract infections and skin and soft tissue infections) and incident cognitive decline and dementia in longitudinal studies. A secondary objective will be to identify gaps in literature and recommendations for future research on this topic.

METHODS

The present systematic review protocol was developed according to the Preferred Reporting Items for Systematic Reviews and Meta-analyses Protocols statement and was registered on the PROSPERO database.[24, 25] Any amendments to the protocol will be updated in PROSPERO.

We will report the systematic review in line with the Preferred Reporting Items for Systematic Reviews and Meta-Analyses statement.[26] If a meta-analysis is feasible, it will be reported according to the Meta-analysis of Observational Studies in Epidemiology statement.[27]

Eligibility criteria

Studies will be eligible for inclusion into the present study if they meet the following inclusion criteria:

Study design

We will include longitudinal studies; retrospective and prospective cohort studies, case control studies and randomised controlled trials (RCTs). We will include studies specifically investigating the association between common bacterial infections and cognitive decline or dementia will be eligible for inclusion. Although it would not be possible to perform randomised controlled trials specifically addressing our research question, we will consider

For peer review only

studies derived from RCTs which could include cohort or case control studies from an RCT data source. We will include studies in which the exposure is ascertained at least 3 months prior to the occurrence of the outcome events in order to investigate the temporal relationship between common bacterial infections and subsequent cognitive decline or dementia.

Study population

We will include human studies of adults aged 18 years and over with no restrictions on the sex, ethnicity, prior health status or residence of participants. We will include studies conducted in any healthcare setting.

Exposure

We will include studies in which exposure is defined as symptomatic illness due to common bacterial infections, either suspected clinically or confirmed by isolation of a bacterial pathogen. Types of bacterial infections will be subdivided into sepsis, lower respiratory tract infections, skin and soft tissue infections and urinary tract infections. The possible pathophysiological mechanisms of bacterial infections on cognitive decline or dementia may differ depending on the site of infection, and thus we will only include studies assessing the independent effect of each type of infection on our outcomes. We will exclude studies that focus on a specific bacterial pathogen as the exposure rather than the symptomatic disease (i.e isolation of a bacterium by polymerase chain reaction (PCR) alone in the absence of clinical symptoms).

Comparators

Studies eligible for inclusion will include a comparison group. For cohort studies and secondary analyses of longitudinal RCT data, individuals exposed to common bacterial infections will be compared to those unexposed to infections. For case control studies, cases with dementia or cognitive decline will be compared to a control group without dementia or cognitive decline.

Outcomes

We will have two primary outcomes. These will be (1) incident cognitive decline and (2) incident dementia (all types). Cognitive decline will be defined clinically. Dementia will also be defined clinically, with or without neuroimaging or histopathology. If a sufficient number of eligible studies are available, dementia will be subdivided into dementia types.

Literature searches

We will systematically search electronic databases of published and grey literature from inception to 18 March 2019. The following databases will be searched: MEDLINE (Ovid interface), EMBASE (Ovid interface), Global health (Ovid interface), PsycINFO (Ovid Interface), Web of Science, Scopus, Cochrane Library, the Cumulative Index to Nursing and Allied Health Literature (CINAHL), Open Grey and the British Library of Electronic Theses databases (ETHOS). Additionally, we will search the reference lists of the included studies to identify any relevant articles not captured in the search strategy. We will search the databases using subject headings, where possible, and keywords related to the exposure, outcome and study design. These search terms will be combined using Boolean logical operators.

For peer review only

We carried out a preliminary search on PubMed to ensure we would capture a sufficient number of studies for inclusion into the review. We developed a search strategy for the MEDLINE database which is provided in online supplementary appendix 1. The search strategy was developed in consultation with a librarian at the London School of Hygiene and Tropical Medicine and was subsequently peer reviewed based on the Peer-Review for Electronic Search Strategies (PRESS).[28] We will translate our search strategy in all databases using search syntaxes specific to each database. No restrictions will be placed on the geographical location, language or date of publication of the studies. Any potentially relevant non-English studies will be translated.

Study records

Data management
We will import the search results into the reference manager software EndNote (X8.0.2). Duplicate entries will be identified and removed.

Study selection
Two researchers will independently screen all titles and abstracts against the eligibility criteria. The researchers will then independently screen the full text articles of potentially eligible articles and decide on whether the inclusion criteria have been met. Any disagreements between the reviewers will be discussed and if necessary a third reviewer will be consulted. Reasons for exclusion of studies will be recorded. We will document the study selection process using the Preferred Reporting Items for Systematic Reviews and Meta-Analyses flow diagram.[26] If multiple papers arise from the same population, we will include the paper with the largest sample size and most detailed information about the exposure and outcome. We pilot tested our study selection process to ensure that the inclusion criteria can be reliably applied.

Data collection process
Two researchers will independently extract data from included papers onto a predesigned form. If necessary, authors will be contacted directly to obtain any missing information. We will perform pilot testing on the data extraction form to identify any missing or irrelevant criteria, and we will modify the form accordingly.

Data items

We will use the Population, Exposure, Comparator, Outcomes and Study characteristics (PECOS) framework to design our data extraction form. The following information will be extracted:

1. Population: age (mean, median or range), sex, inclusion and exclusion criteria.

2. Exposure: definition of exposure, type of bacterial infection, number of exposed.

3. Comparators: identification and definition of comparator, number of comparators.

For peer review only

4. Outcomes: definition of outcome and identification of cognitive decline and dementia, number of participants with the outcome.

5. Study Characteristics: authors, name of study, year of publication, study design, type of longitudinal study, healthcare setting, country, sample size, duration of follow up.

Regarding the study results, we will extract unadjusted and adjusted estimates and their corresponding 95% CI for each exposure and outcome. Additionally, we will extract data on the confounding variables adjusted for in each study as well as data on sub-group or sensitivity analyses. As part of our risk of bias and study quality assessment, we will assess whether studies have adequately adjusted for confounding factors. Factors considered to be potential confounders include age,[29] sex,[30] socioeconomic status,[31] ethnicity,[32] smoking,[33] alcohol consumption,[34] cardiovascular disease,[35] diabetes,[36] renal dysfunction,[37] psychiatric disorders,[38] cerebrovascular disease,[39] chronic obstructive pulmonary disease,[40] and immunodeficiency disorders. [41] We will assess whether studies have adequately assessed for potential confounders as part of our risk of bias and study quality assessments.

Risk of bias in individual studies

We will use a sample of studies to pilot test the risk of bias form to ensure the criteria can be applied consistently by both reviewers. Two researchers will independently assess the risk of bias in line with the Cochrane Collaboration approach.[42, 43] We will examine the risk of bias for randomised controlled trials using the following domains: sequence generation; allocation concealment; blinding of participants and personnel; blinding of outcome assessment; incomplete outcome data; selective outcome reporting and other potential threats to validity.[43] We will assess the risk of bias for observational studies using the following domains: confounding, selection of participants, misclassification of variables, missing data, reverse causation, generalisability and study power.

Data synthesis and meta-bias(es)

We will group studies according to the outcome (cognitive decline or dementia) and exposure (type of common bacterial infection) and synthesise them narratively. Data will be summarised in pre-defined tables. We will consider subgroup analyses according to age-group, gender, dementia subtype and risk of bias.

A meta-analysis will be considered feasible if there are a minimum of at least two studies that are homogeneous in terms of study design, type of common bacterial infection and type of outcome. We will pool effect measures (odds ratios, risk ratios or hazard ratios) from the studies in order to perform the meta-analysis.

We will assess statistical heterogeneity through the use of forests plots, chi-squared tests and I^2 statistic. Depending on the level of heterogeneity, a fixed or random effects model will be selected to calculate the pooled incidence and corresponding 95% CI. A chi square test with a p value of 0.1 will be considered statistically significant. We will consider an I^2 value of >25% to indicate moderate heterogeneity, which will guide the use of a random effects model.[44, 45] We will assess publication bias and small study effects using funnel

For peer review only

plots, provided that there are 10 or more studies eligible for inclusion into the meta-analysis.[46]

Confidence in cumulative evidence

We will assess the overall quality of evidence for each outcome using the Grading of Recommendations, Assessment, Development and Evaluation tool.[47] The domains assessed will include study limitations, inconsistency, indirectness, imprecision and publication bias.[48] The strength of the evidence will be categorised as high, moderate, low and very low.

Patient and public involvement

No patients or public were directly involved in the design of this study. However, we sought advice on the dissemination of our findings from lay volunteers assigned to Rutendo Muzambi's PhD studentship by the Alzheimer's Society.

ETHICS AND DISSEMINATION

This systematic review will provide evidence for the role of common bacterial infections in the development of cognitive decline and dementia. The systematic review will be submitted for publication in a peer-reviewed journal and the results may be presented at national and international conferences and meetings relevant to the field. This review will highlight gaps in current literature and identify future research directions. Ethical approval will not be required as the review will summarise data from previous studies.

Author contribution

CWG, KB and LS, conceived and designed the study, revised the protocol and search strategy critically. RM conceived and designed the study, drafted and revised the protocol, and developed and revised the search strategy. CB contributed to the design of the study and revised the protocol and search strategy critically. The final version of the protocol was read and approved by all authors. We would like to thank the Alzheimer's Society research volunteers for their advice on the dissemination of our findings.

Funding

Rutendo Muzambi is supported by an Alzheimer's Society PhD studentship 379 (AS-PhD-17-013). The funder played no role in developing the protocol.

Competing interests

No conflict of interest was reported by any of the authors.

For peer review only

References

1. Sousa RM, Ferri CP, Acosta D, et al. Contribution of chronic diseases to disability in elderly people in countries with low and middle incomes: a 10/66 Dementia Research Group population-based survey. *Lancet* 2009;374:1821-30.
2. Harwood RH, Sayer AA, Hirschfeld M. Current and future worldwide prevalence of dependency, its relationship to total population, and dependency ratios. *Bull World Health Organ* 2004;82:251-8.
3. Global, regional, and national burden of Alzheimer's disease and other dementias, 1990-2016: a systematic analysis for the Global Burden of Disease Study 2016. *Lancet Neurol* 2019;18:88-106.
4. Organization. WH. Global action plan on the public health response to dementia 2017-2025. Geneva: World Health Organization; 2017.
5. Prince M, Guerchet M, Prina M. Policy Brief for Heads of Government: The Global Impact of Dementia 2013–2050. London: Alzheimer's Disease International 2013 [[Internet] http://www.alz.co.uk/research/G8-policy-brief (Last accessed on 25 Jan 2019)
6. Hugo J, Ganguli M. Dementia and cognitive impairment: epidemiology, diagnosis, and treatment. *Clin Geriatr Med* 2014;30:421-42.
7. Matthews FE, Stephan BC, Robinson L, et al. A two decade dementia incidence comparison from the Cognitive Function and Ageing Studies I and II. *Nat Commun* 2016;7:11398.
8. Organization. WH. Dementia: a public health priority 2012 [[Internet] http://www.who.int/mental_health/publications/dementia_report_2012/en/ (Last accessed on 6 Feb 2019)
9. Barnes DE, Yaffe K. The projected effect of risk factor reduction on Alzheimer's disease prevalence. *Lancet Neurol* 2011;10:819-28.
10. Norton S, Matthews FE, Barnes DE, et al. Potential for primary prevention of Alzheimer's disease: an analysis of population-based data. *Lancet Neurol* 2014;13:788-94.
11. Livingston G, Sommerlad A, Orgeta V, et al. Dementia prevention, intervention, and care. *Lancet* 2017;390:2673-734.
12. Sochocka M, Zwolinska K, Leszek J. The Infectious Etiology of Alzheimer's Disease. *Curr Neuropharmacol* 2017;15:996-1009.
13. Lim SL, Rodriguez-Ortiz CJ, Kitazawa M. Infection, systemic inflammation, and Alzheimer's disease. *Microbes Infect* 2015;17:549-56.
14. Mawanda F, Wallace R. Can Infections Cause Alzheimer's Disease? *Epidemiol Rev* 2013;35:161-80.
15. Maheshwari P, Eslick GD. Bacterial infection and Alzheimer's disease: a meta-analysis. *J Alzheimers Dis* 2015;43:957-66.
16. van Gool WA, van de Beek D, Eikelenboom P. Systemic infection and delirium: when cytokines and acetylcholine collide. *Lancet* 2010;375:773-5.
17. Girard TD, Jackson JC, Pandharipande PP, et al. Delirium as a predictor of long-term cognitive impairment in survivors of critical illness. *Crit Care Med* 2010;38:1513-20.
18. MacLullich AM, Beaglehole A, Hall RJ, et al. Delirium and long-term cognitive impairment. *Int Rev Psychiatry* 2009;21:30-42.
19. Davis DH, Muniz Terrera G, Keage H, et al. Delirium is a strong risk factor for dementia in the oldest-old: a population-based cohort study. *Brain* 2012;135:2809-16.

For peer review only

20. Foley NC, Affoo RH, Martin RE. A Systematic Review and Meta-Analysis Examining Pneumonia-Associated Mortality in Dementia. *Dement Geriatr Cogn Disord* 2015;39:52-67.
21. Chae JH, Miller BJ. Beyond Urinary Tract Infections (UTIs) and Delirium: A Systematic Review of UTIs and Neuropsychiatric Disorders. *J Psychiatr Pract* 2015;21:402-11.
22. Fulop T, Witkowski JM, Bourgade K, et al. Can an Infection Hypothesis Explain the Beta Amyloid Hypothesis of Alzheimer's Disease? *Front Aging Neurosci* 2018;10:224.
23. Itzhaki RF, Lathe R, Balin BJ, et al. Microbes and Alzheimer's Disease. *Journal of Alzheimer's disease : JAD* 2016;51:979-84.
24. Moher D, Shamseer L, Clarke M, et al. Preferred reporting items for systematic review and meta-analysis protocols (PRISMA-P) 2015 statement. *Syst Rev* 2015;4:1.
25. Booth A, Clarke M, Dooley G, et al. The nuts and bolts of PROSPERO: an international prospective register of systematic reviews. *Syst Rev* 2012;1:2.
26. Moher D, Liberati A, Tetzlaff J, et al. Preferred reporting items for systematic reviews and meta-analyses: the PRISMA statement. *PLoS Med* 2009;6:e10000097.
27. Stroup DF, Berlin JA, Morton SC, et al. Meta-analysis of observational studies in epidemiology: a proposal for reporting. Meta-analysis Of Observational Studies in Epidemiology (MOOSE) group. *Jama* 2000;283:2008-12.
28. McGowan J, Sampson M, Salzwedel DM, et al. PRESS Peer Review of Electronic Search Strategies: 2015 Guideline Statement. *J Clin Epidemiol* 2016;75:40-6.
29. Matthews F, Brayne C, Medical Research Council Cognitive F, et al. The incidence of dementia in England and Wales: findings from the five identical sites of the MRC CFA Study. *PLoS Med* 2005;2:e193-e.
30. Andersen K, Launer LJ, Dewey ME, et al. Gender differences in the incidence of AD and vascular dementia: The EURODEM Studies. EURODEM Incidence Research Group. *Neurology* 1999;53:1992-7.
31. Karp A, Kareholt I, Qiu C, et al. Relation of education and occupation-based socioeconomic status to incident Alzheimer's disease. *Am J Epidemiol* 2004;159:175-83.
32. Tang MX, Cross P, Andrews H, et al. Incidence of AD in African-Americans, Caribbean Hispanics, and Caucasians in northern Manhattan. *Neurology* 2001;56:49-56.
33. Zhong G, Wang Y, Zhang Y, et al. Smoking is associated with an increased risk of dementia: a meta-analysis of prospective cohort studies with investigation of potential effect modifiers. *PloS One* 2015;10:e0118333-e.
34. Sabia S, Fayosse A, Dumurgier J, et al. Alcohol consumption and risk of dementia: 23 year follow-up of Whitehall II cohort study. *BMJ* 2018;362:k2927.
35. Justin BN, Turek M, Hakim AM. Heart disease as a risk factor for dementia. *Clin Epidemiol* 2013;5:135-45.
36. Gudala K, Bansal D, Schifano F, et al. Diabetes mellitus and risk of dementia: A meta-analysis of prospective observational studies. *J Diabetes Investig* 2013;4:640-50.
37. Deckers K, Camerino I, van Boxtel MPJ, et al. Dementia risk in renal dysfunction: A systematic review and meta-analysis of prospective studies. *Neurology* 2017;88:198-208.
38. Zilkens RR, Bruce DG, Duke J, et al. Severe psychiatric disorders in mid-life and risk of dementia in late- life (age 65-84 years): a population based case-control study. *Curr Alzheimer Res* 2014;11:681-93.
39. Kuźma E, Lourida I, Moore SF, et al. Stroke and dementia risk: A systematic review and meta-analysis. *Alzheimers Dement* 2018;14:1416-26.
40. Dodd JW. Lung disease as a determinant of cognitive decline and dementia. *Alzheimers Res Ther* 2015;7:32.

For peer review only

41. Bhaskaran K, Mussini C, Antinori A, et al. Changes in the incidence and predictors of human immunodeficiency virus–associated dementia in the era of highly active antiretroviral therapy. *Ann Neurol* 2008;63:213-21.

42. Sterne JA, Hernán MA, Reeves BC, et al. ROBINS-I: a tool for assessing risk of bias in non-randomised studies of interventions. *BMJ* 2016;355.

43. Higgins JPT, Altman DG, Gøtzsche PC, et al. The Cochrane Collaboration's tool for assessing risk of bias in randomised trials. *BMJ* 2011;343.

44. Riley RD, Higgins JPT, Deeks JJ. Interpretation of random effects meta-analyses. *BMJ* 2011;342.

45. Higgins J, Green S. Section 9.5.2. Identifying and measuring heterogeneity in Cochrane handbook for systematic reviews of interventions 2011 [[Internet] https://handbook-5-1.cochrane.org/chapter_9/9_5_2_identifying_and_measuring_heterogeneity.htm; (Last accessed on 8 Mar 2019)

46. Sterne JA, Egger M. Funnel plots for detecting bias in meta-analysis: guidelines on choice of axis. *J Clin Epidemiol* 2001;54:1046-55.

47. Guyatt GH, Oxman AD, Vist GE, et al. GRADE: an emerging consensus on rating quality of evidence and strength of recommendations. *BMJ* 2008;336:924-6.

48. Balshem H, Helfand M, Schünemann HJ, et al. GRADE guidelines: 3. Rating the quality of evidence. *J Clin Epidemiol* 2011;64:401-6.

For peer review only

Appendix 1. Medline (OVID) search strategy

1. Pneumonia/ or pneumonia, bacterial/
2. Pneumonia.ti,ab
3. Lower respiratory tract infection*.ti,ab
4. (LRTI or LRTIS).ti,ab.
5. Exp urinary tract infections/
6. (Urinary adj5 infection*).ti,ab.
7. (UTI or UTIS).ti,ab
8. exp Cystitis/
9. (bacteriuria or pyuria or cystitis or pyelonephritis or cellulitis).ti,ab.
10. exp cellulitis/
11. (Skin and soft tissue infection).mp.
12. Exp sepsis/
13. (septic* or sepsis or septic?emia or systemic inflammatory response syndrome or blood stream infection or py?emia).ti,ab.
14. 1 or 2 or 3 or 4 or 5 or 6 or 7 or 8 or 9 or 10 or 11 or 12 or 13
15. Exp dementia/
16. Exp prion diseases/
17. (huntington* or kluver-bucy or prion disease or Creutzfeldt-jakob or primary progressive aphasia).ti,ab
18. (Dement* or Alzheimer*).ti,ab
19. (Lewy*adj2 bod*).ti,ab.
20. Cognitive dysfunction/
21. (Mild cognitive impairment or MCI).ti,ab
22. ((cognit* or memory or cerebr* or mental*) adj3 (declin* or impair* or los* or deteriorat* or degenerat* or complain* or disturb* or disorder*)).ti,ab.
23. Cognitive function.ti,ab.
24. 15 or 16 or 17 or 18 or 19 or 20 or 21 or 22 or 23
25. cohort studies/ or longitudinal study/ or follow-up study/ or prospective study/ or retrospective study/ or cohort.ti,ab. or longitudinal.ti,ab. or prospective.ti,ab. or retrospective.ti,ab.
26. Case-Control Studies/ or Control Groups/ or Matched-Pair Analysis/ or ((case* adj5 control*) or (case adj3 comparison*) or control group*).ti,ab.
27. Incidence/ or incidence.ti,ab,kw.
28. (hazard ratio or HR or odds ratio or relative risk or RR).ti,ab.
29. 25 or 26 or 27 or 28
30. 14 and 24 and 29

For peer review only

Reporting checklist for protocol of a systematic review.

Based on the PRISMA-P guidelines.

Instructions to authors

Complete this checklist by entering the page numbers from your manuscript where readers will find each of the items listed below.

Your article may not currently address all the items on the checklist. Please modify your text to include the missing information. If you are certain that an item does not apply, please write "n/a" and provide a short explanation.

Upload your completed checklist as an extra file when you submit to a journal.

In your methods section, say that you used the PRISMA-Preporting guidelines, and cite them as:

Moher D, Shamseer L, Clarke M, Ghersi D, Liberati A, Petticrew M, Shekelle P, Stewart LA. Preferred Reporting Items for Systematic Review and Meta-Analysis Protocols (PRISMA-P) 2015 statement. Syst Rev. 2015;4(1):1.

		Reporting Item	Page Number
**Title**			
Identification	#1a	Identify the report as a protocol of a systematic review	1
Update	#1b	If the protocol is for an update of a previous systematic review, identify as such	n/a
**Registration**			
	#2	If registered, provide the name of the registry (such as PROSPERO) and registration number	2
**Authors**			
Contact	#3a	Provide name, institutional affiliation, e-mail address of all protocol authors; provide physical mailing address of corresponding author	1
Contribution	#3b	Describe contributions of protocol authors and identify the guarantor of the review	9
**Amendments**			

For peer review only

	#4	If the protocol represents an amendment of a previously completed or published protocol, identify as such and list changes; otherwise, state plan for documenting important protocol amendments	n/a
**Support**			
Sources	#5a	Indicate sources of financial or other support for the review	9
Sponsor	#5b	Provide name for the review funder and / or sponsor	9
Role of sponsor or funder	#5c	Describe roles of funder(s), sponsor(s), and / or institution(s), if any, in developing the protocol	9
**Introduction**			
Rationale	#6	Describe the rationale for the review in the context of what is already known	4/5
Objectives	#7	Provide an explicit statement of the question(s) the review will address with reference to participants, interventions, comparators, and outcomes (PICO)	5
**Methods**			
Eligibility criteria	#8	Specify the study characteristics (such as PICO, study design, setting, time frame) and report characteristics (such as years considered, language, publication status) to be used as criteria for eligibility for the review	5/6
Information sources	#9	Describe all intended information sources (such as electronic databases, contact with study authors, trial registers or other grey literature sources) with planned dates of coverage	6/7
Search strategy	#10	Present draft of search strategy to be used for at least one electronic database, including planned limits, such that it could be repeated	7 (and in appendices))
Study records - data management	#11a	Describe the mechanism(s) that will be used to manage records and data throughout the review	7
Study records - selection process	#11b	State the process that will be used for selecting studies (such as two independent reviewers) through each phase of the review (that is, screening, eligibility and inclusion in meta-analysis)	7

For peer review only

Study records - data collection process	#11c	Describe planned method of extracting data from reports (such as piloting forms, done independently, in duplicate), any processes for obtaining and confirming data from investigators	7
Data items	#12	List and define all variables for which data will be sought (such as PICO items, funding sources), any pre-planned data assumptions and simplifications	7/8
Outcomes and prioritization	#13	List and define all outcomes for which data will be sought, including prioritization of main and additional outcomes, with rationale	6
Risk of bias in individual studies	#14	Describe anticipated methods for assessing risk of bias of individual studies, including whether this will be done at the outcome or study level, or both; state how this information will be used in data synthesis	8
Data synthesis	#15a	Describe criteria under which study data will be quantitatively synthesised	8
Data synthesis	#15b	If data are appropriate for quantitative synthesis, describe planned summary measures, methods of handling data and methods of combining data from studies, including any planned exploration of consistency (such as I2, Kendall's τ)	8
Data synthesis	#15c	Describe any proposed additional analyses (such as sensitivity or subgroup analyses, meta-regression)	8
Data synthesis	#15d	If quantitative synthesis is not appropriate, describe the type of summary planned	8
Meta-bias(es)	#16	Specify any planned assessment of meta-bias(es) (such as publication bias across studies, selective reporting within studies)	9
Confidence in cumulative evidence	#17	Describe how the strength of the body of evidence will be assessed (such as GRADE)	9

Notes:

- 10: 7 (and in appendices)) The PRISMA-P checklist is distributed under the terms of the Creative Commons Attribution License CC-BY 4.0. This checklist was completed on 09. June 2019 using https://www.goodreports.org/, a tool made by the EQUATOR Network in collaboration with Penelope.ai

For peer review only

BMJ Open

Common bacterial infections and risk of incident cognitive decline or dementia: a systematic review protocol

Journal:	*BMJ Open*
Manuscript ID	bmjopen-2019-030874.R2
Article Type:	Protocol
Date Submitted by the Author:	09-Aug-2019
Complete List of Authors:	Muzambi, Rutendo; London School of Hygiene and Tropical Medicine, Non-communicable disease epidemiology<br>Bhaskaran, Krishnan; LSHTM, NCDE<br>Brayne, Carol; University of Cambridge, Institute of Public Health<br>Smeeth, Liam; London School of Hygiene and Tropical Medicine, Epidemiology and Population Health<br>Warren-Gash, Charlotte; London School of Hygiene and Tropical Medicine, Non-communicable disease epidemiology
**Primary Subject Heading**:	Epidemiology
Secondary Subject Heading:	Neurology, Infectious diseases
Keywords:	Dementia < NEUROLOGY, INFECTIOUS DISEASES, EPIDEMIOLOGY, Epidemiology < INFECTIOUS DISEASES

SCHOLARONE™
Manuscripts

Common bacterial infections and risk of incident cognitive decline or dementia: a systematic review protocol

Authors: Rutendo Muzambi[1], Krishnan Bhaskaran[1], Carol Brayne[2], Liam Smeeth[1] Charlotte Warren-Gash[1]

Author affiliations:

1. Faculty of Epidemiology and Population Health, London School of Hygiene and tropical, London, WC1E 7HT, UK
2. Cambridge Institute of Public Health, Cambridge University, Cambridge, United Kingdom

Correspondence to:
Rutendo Muzambi. Email: Rutendo.muzambi@lshtm.ac.uk. Tel: 020 7299 4678

Word Count: 2645 excluding title page, abstract, references and tables.

For peer review only

ABSTRACT

Introduction The global burden of dementia is rising, emphasising the urgent need to develop effective approaches to risk reduction. Recent evidence suggests that common bacterial infections may increase the risk of dementia, however the magnitude and timing of the association as well as the patient groups affected remains unclear. We will review existing evidence of the association between common bacterial infections and incident cognitive decline or dementia.

Methods and analysis We will conduct a comprehensive search of published and grey literature from inception to 18 March 2019. The following electronic databases will be searched; MEDLINE, EMBASE, Global health, PsycINFO, Web of Science, Scopus, Cochrane Library, the Cumulative Index to Nursing and Allied Health Literature (CINAHL), Open Grey and the British Library of electronic theses databases (ETHOS). There will be no restrictions on the date, language or geographical location of the studies. We will include longitudinal studies with a common clinically symptomatic bacterial infection as an exposure and incident cognitive decline or dementia as an outcome. Study selection, data extraction and risk of bias will be performed independently by two researchers. We will assess the risk of bias using the Cochrane collaboration approach. The overall quality of the studies will be assessed using the Grading of Recommendations, Assessment, Development and Evaluations criteria. We will explore the heterogeneity of relevant studies and, if feasible, a meta-analysis will be performed, otherwise we will present a narrative synthesis. We will group the results by exposure and outcome definitions and differences will be described by subgroups and outcomes.

Ethics and dissemination Ethical approval will not be required as this is a systematic review of existing research in the public domain. Results will be disseminated in a peer-reviewed journal and presented at national and international meetings and conferences.

PROSPERO registration number CRD42018119294

Keywords dementia, Alzheimer's disease, bacterial infections, systematic review protocol

For peer review only

ARTICLE SUMMARY

Strengths and limitations

- To the best of our knowledge, this will be the first systematic review assessing the association between common clinically symptomatic bacterial infections and the risk of incident cognitive decline and dementia in longitudinal studies.
- We will perform a comprehensive search of published and grey literature with no restrictions on date, language or geographical location.
- The review will develop existing evidence to generate better knowledge on the magnitude and direction of the relationship between common bacterial infections and subsequent cognitive decline or dementia.
- Heterogeneity in the way infections, cognitive decline and dementia are defined is expected, which could affect the feasibility of performing a meta-analysis and interpretation of findings.
- There may be difficulty ascertaining whether lower respiratory tract infections are bacterial or viral.

For peer review only

For peer review only - http://bmjopen.bmj.com/site/about/guidelines.xhtml

INTRODUCTION

Rationale

Dementia is a clinical syndrome that significantly contributes to disability and dependence worldwide, with a devastating impact on individuals, caregivers and healthcare services.[1-3] It is characterised by a progressive deterioration in cognition and behaviour that interferes with an individual's ability to perform activities of daily living.[4] In 2018, approximately 50 million people worldwide were estimated to be living with dementia, and this figure is projected to rise to 152 million by 2050.[5]

Age is the single biggest risk factor for dementia, with the risk doubling every 5 years after the age of 65.[6] Despite this, there have been some indications that the risk for dementia can be reduced, with more recent cohorts in the UK demonstrating a significantly lower risk at any given age.[7] It has become clear from clinical and neuropathological studies that the risk for dementia is complex, not driven by genetics, vascular factors or age alone. Given the rapidly increasing ageing population and the absence of pharmacological treatments that can delay the onset or progression of dementia, identification of effective strategies to reduce risk, as age increases, has become a public health priority.[8] As a result, recent literature has focused on modifiable risk factors. It has been estimated that around a third of dementia cases worldwide can be attributed to modifiable risk factors.[9-11]

Bacterial infections have been identified as potential risk factors for dementia. For the past few decades, a large body of research has been published on the association between bacterial pathogens and Alzheimer's disease, particularly in post mortem brain tissue.[12-14] Despite this, the temporality of this relationship remains unclear due to the cross-sectional nature of the data collected in these studies. A recent meta-analysis of 27 serological, cerebrospinal fluid and post-mortem brain studies found that chlamydia pneumonia and spirochaetal infection were associated with a five-fold (OR 5.66; 95% CI:1.83-17.51) and ten-fold (OR 10.61; 95% CI: 3.38-33.29) increased risk of Alzheimer's disease, respectively.[15] However, temporality could not be assessed in these studies. The meta-analysis was further limited by the inclusion of studies with small sample sizes and the focus on Alzheimer's disease rather than all types of dementia.

Common bacterial infections are well recognised to be associated with acute changes in cognition, manifested as delirium, among older adults.[16] In turn, delirium is strongly associated with an increased risk of subsequent cognitive decline and dementia.[17-19] This raises the challenge of disentangling the relative contribution of the (potentially reversible) ageing immune system's response to acute infections, from ongoing neuropathological processes, both of which may affect cognition.

Longitudinal studies with a follow up time sufficient enough for delirium to resolve, are important in distinguishing between delirium and long-term cognitive impairment. Additionally, longitudinal studies that compare incidence of cognitive decline or dementia in individuals with and without common bacterial infections provide evidence for the temporality and magnitude of this association. Although, the prevalence of common bacterial infections, such as pneumonia and urinary tract infections, in individuals with

For peer review only - http://bmjopen.bmj.com/site/about/guidelines.xhtml

dementia is well known,[20, 21] the incidence of cognitive decline and dementia is less established.

The mechanisms by which bacterial infections may increase the risk of cognitive decline and dementia are unclear but may involve inflammation.[22, 23] Bacterial infections may trigger an inflammatory response in the brain resulting in the release of pro-inflammatory mediators and activation of cytotoxic microglia. This may result in deterioration of cognitive function, possibly increasing the risk of dementia.[12]

To date, no systematic review has been performed on the incidence of cognitive decline and dementia in individuals with a common bacterial infection causing clinical illness. As these infections frequently occur in the community, early recognition, treatment or prevention of common bacterial infections could have important public health implications in reducing the burden of dementia.

Objectives

The primary objective of the proposed systematic review is to summarise the current literature investigating the association between common clinically symptomatic bacterial infections (sepsis, lower respiratory tract infections, urinary tract infections and skin and soft tissue infections) and incident cognitive decline and dementia in longitudinal studies. A secondary objective will be to identify gaps in literature and recommendations for future research on this topic.

METHODS

The present systematic review protocol was developed according to the Preferred Reporting Items for Systematic Reviews and Meta-analyses Protocols statement and was registered on the PROSPERO database.[24, 25] Any amendments to the protocol will be updated in PROSPERO.

We will report the systematic review in line with the Preferred Reporting Items for Systematic Reviews and Meta-Analyses statement.[26] If a meta-analysis is feasible, it will be reported according to the Meta-analysis of Observational Studies in Epidemiology statement.[27]

Eligibility criteria

Studies will be eligible for inclusion into the present study if they meet the following inclusion criteria:

Study design

We will include longitudinal studies; retrospective and prospective cohort studies, case control studies and randomised controlled trials (RCTs). We will include studies specifically investigating the association between common bacterial infections and cognitive decline or dementia will be eligible for inclusion. Although it would not be possible to perform randomised controlled trials specifically addressing our research question, we will consider

For peer review only

For peer review only - http://bmjopen.bmj.com/site/about/guidelines.xhtml

studies derived from RCTs which could include cohort or case control studies from an RCT data source. We will include studies in which the exposure is ascertained prior to the occurrence of the outcome events in order to investigate the temporal relationship between common bacterial infections and subsequent cognitive decline or dementia. Additionally, to avoid including studies focusing on short term reversible changes in cognition, rather than long term cognitive decline, we will include studies in which cognitive decline was measured at least 3 months following infection.

Study population
We will include human studies of adults aged 18 years and over with no restrictions on the sex, ethnicity, prior health status or residence of participants. We will include studies conducted in any healthcare setting.

Exposure
We will include studies in which exposure is defined as symptomatic illness due to common bacterial infections, either suspected clinically or confirmed by isolation of a bacterial pathogen. Types of bacterial infections will be subdivided into sepsis, lower respiratory tract infections, skin and soft tissue infections and urinary tract infections. The possible pathophysiological mechanisms of bacterial infections on cognitive decline or dementia may differ depending on the site of infection, and thus we will only include studies assessing the independent effect of each type of infection on our outcomes. In addition, infections are likely to differ in terms of severity, particularly sepsis, which further highlights the need to assess their effect separately. We will exclude studies that focus on a specific bacterial pathogen as the exposure rather than the symptomatic disease (i.e isolation of a bacterium by polymerase chain reaction (PCR) alone in the absence of clinical symptoms).

Comparators
Studies eligible for inclusion will include a comparison group. For cohort studies and secondary analyses of longitudinal RCT data, individuals exposed to common bacterial infections will be compared to those unexposed to infections. For case control studies, cases with dementia or cognitive decline will be compared to a control group without dementia or cognitive decline.

Outcomes
We will have two primary outcomes. These will be (1) incident cognitive decline and (2) incident dementia (all types). Cognitive decline will be defined clinically. Dementia will also be defined clinically, with or without neuroimaging or histopathology. If a sufficient number of eligible studies are available, dementia will be subdivided into dementia types.

Literature searches
We will systematically search electronic databases of published and grey literature from inception to 18 March 2019. The following databases will be searched: MEDLINE (Ovid interface), EMBASE (Ovid interface), Global health (Ovid interface), PsycINFO (Ovid Interface), Web of Science, Scopus, Cochrane Library, the Cumulative Index to Nursing and Allied Health Literature (CINAHL), Open Grey and the British Library of Electronic Theses databases (ETHOS). Additionally, we will search the reference lists of the included studies to identify any relevant articles not captured in the search strategy. We will search the

For peer review only - http://bmjopen.bmj.com/site/about/guidelines.xhtml

databases using subject headings, where possible, and keywords related to the exposure, outcome and study design. These search terms will be combined using Boolean logical operators.

We carried out a preliminary search on PubMed to ensure we would capture a sufficient number of studies for inclusion into the review. We developed a search strategy for the MEDLINE database which is provided in online supplementary appendix 1. The search strategy was developed in consultation with a librarian at the London School of Hygiene and Tropical Medicine and was subsequently peer reviewed based on the Peer-Review for Electronic Search Strategies (PRESS).[28] We will translate our search strategy in all databases using search syntaxes specific to each database. No restrictions will be placed on the geographical location, language or date of publication of the studies. Any potentially relevant non-English studies will be translated.

Study records

Data management
We will import the search results into the reference manager software EndNote (X8.0.2). Duplicate entries will be identified and removed.

Study selection
Two researchers will independently screen all titles and abstracts against the eligibility criteria. The researchers will then independently screen the full text articles of potentially eligible articles and decide on whether the inclusion criteria have been met. Any disagreements between the reviewers will be discussed and if necessary a third reviewer will be consulted. Reasons for exclusion of studies will be recorded. We will document the study selection process using the Preferred Reporting Items for Systematic Reviews and Meta-Analyses flow diagram.[26] If multiple papers arise from the same population, we will include the paper with the largest sample size and most detailed information about the exposure and outcome. We pilot tested our study selection process to ensure that the inclusion criteria can be reliably applied.

Data collection process
Two researchers will independently extract data from included papers onto a predesigned form. If necessary, authors will be contacted directly to obtain any missing information. We will perform pilot testing on the data extraction form to identify any missing or irrelevant criteria, and we will modify the form accordingly.

Data items

We will use the Population, Exposure, Comparator, Outcomes and Study characteristics (PECOS) framework to design our data extraction form. The following information will be extracted:

1. Population: age (mean, median or range), sex, inclusion and exclusion criteria.

For peer review only

2. Exposure: definition of exposure, type of bacterial infection, cause of sepsis, number of exposed.

3. Comparators: identification and definition of comparator, number of comparators.

4. Outcomes: definition of outcome and identification of cognitive decline and dementia, number of participants with the outcome.

5. Study Characteristics: authors, name of study, year of publication, study design, type of longitudinal study, healthcare setting, country, sample size, duration of follow up.

Regarding the study results, we will extract unadjusted and adjusted estimates and their corresponding 95% CI for each exposure and outcome. Data on sub-group or sensitivity analyses will be extracted. We will extract additional data on antibiotic treatment, if indicated, given that antibiotics have been associated with delirium,[29] which is in turn a risk factor for cognitive decline and dementia. We will also extract data on confounding variables. Factors considered to be potential confounders include age,[30] sex,[31] socioeconomic status,[32] ethnicity,[33] smoking,[34] alcohol consumption,[35] cardiovascular disease,[36] diabetes,[37] renal dysfunction,[38] psychiatric disorders,[39] cerebrovascular disease,[40] chronic obstructive pulmonary disease,[41] and immunodeficiency disorders. [42] We will assess whether studies have adequately assessed for potential confounders as part of our risk of bias and study quality assessments.

Risk of bias in individual studies

We will use a sample of studies to pilot test the risk of bias form to ensure the criteria can be applied consistently by both reviewers. Two researchers will independently assess the risk of bias in line with the Cochrane Collaboration approach.[43, 44] We will examine the risk of bias for randomised controlled trials using the following domains: sequence generation; allocation concealment; blinding of participants and personnel; blinding of outcome assessment; incomplete outcome data; selective outcome reporting and other potential threats to validity.[44] We will assess the risk of bias for observational studies using the following domains: confounding, selection of participants, misclassification of variables, missing data, reverse causation, generalisability and study power.

Data synthesis and meta-bias(es)

We will group studies according to the outcome (cognitive decline or dementia) and exposure (type of common bacterial infection) and synthesise them narratively. Data will be summarised in pre-defined tables. We will consider subgroup analyses according to age-group, gender, dementia subtype and risk of bias. If possible, we will explore the effect of antibiotic treatment on cognitive decline or dementia.

A meta-analysis will be considered feasible if there are a minimum of at least two studies that are homogeneous in terms of study design, type of common bacterial infection and type of outcome. We will pool effect measures (odds ratios, risk ratios or hazard ratios) from the studies in order to perform the meta-analysis.

For peer review only - http://bmjopen.bmj.com/site/about/guidelines.xhtml

We will assess statistical heterogeneity through the use of forests plots, chi-squared tests and I^2 statistic. Depending on the level of heterogeneity, a fixed or random effects model will be selected to calculate the pooled incidence and corresponding 95% CI. A chi square test with a p value of 0.1 will be considered statistically significant. We will consider an I^2 value of >25% to indicate moderate heterogeneity, which will guide the use of a random effects model.[45, 46] We will assess publication bias and small study effects using funnel plots, provided that there are 10 or more studies eligible for inclusion into the meta-analysis.[47]

Confidence in cumulative evidence

We will assess the overall quality of evidence for each outcome using the Grading of Recommendations, Assessment, Development and Evaluation tool.[48] The domains assessed will include study limitations, inconsistency, indirectness, imprecision and publication bias.[49] The strength of the evidence will be categorised as high, moderate, low and very low.

Patient and public involvement

No patients or public were directly involved in the design of this study. However, we sought advice on the dissemination of our findings from lay volunteers assigned to Rutendo Muzambi's PhD studentship by the Alzheimer's Society.

ETHICS AND DISSEMINATION

This systematic review will provide evidence for the role of common bacterial infections in the development of cognitive decline and dementia. The systematic review will be submitted for publication in a peer-reviewed journal and the results may be presented at national and international conferences and meetings relevant to the field. This review will highlight gaps in current literature and identify future research directions. Ethical approval will not be required as the review will summarise data from previous studies.

Author contribution

CWG, KB and LS, conceived and designed the study, revised the protocol and search strategy critically. RM conceived and designed the study, drafted and revised the protocol, and developed and revised the search strategy. CB contributed to the design of the study and revised the protocol and search strategy critically. The final version of the protocol was read and approved by all authors. We would like to thank the Alzheimer's Society research volunteers for their advice on the dissemination of our findings.

Funding

Rutendo Muzambi is supported by an Alzheimer's Society PhD studentship 379 (AS-PhD-17-013). The funder played no role in developing the protocol.

Competing interests

For peer review only

No conflict of interest was reported by any of the authors.

For peer review only

For peer review only - http://bmjopen.bmj.com/site/about/guidelines.xhtml

References

1. Sousa RM, Ferri CP, Acosta D, et al. Contribution of chronic diseases to disability in elderly people in countries with low and middle incomes: a 10/66 Dementia Research Group population-based survey. *Lancet* 2009;374:1821-30.
2. Harwood RH, Sayer AA, Hirschfeld M. Current and future worldwide prevalence of dependency, its relationship to total population, and dependency ratios. *Bull World Health Organ* 2004;82:251-8.
3. Global, regional, and national burden of Alzheimer's disease and other dementias, 1990-2016: a systematic analysis for the Global Burden of Disease Study 2016. *Lancet Neurol* 2019;18:88-106.
4. Organization. WH. Global action plan on the public health response to dementia 2017-2025. Geneva: World Health Organization; 2017.
5. Patterson C. World Alzheimer report 2018: *The state of the art of dementia research: New frontiers*. London: Alzheimer's Disease International; 2018.
6. Hugo J, Ganguli M. Dementia and cognitive impairment: epidemiology, diagnosis, and treatment. *Clin Geriatr Med* 2014;30:421-42.
7. Matthews FE, Stephan BC, Robinson L, et al. A two decade dementia incidence comparison from the Cognitive Function and Ageing Studies I and II. *Nat Commun* 2016;7:11398.
8. Organization. WH. Dementia: a public health priority 2012 [Internet] http://www.who.int/mental_health/publications/dementia_report_2012/en/ (Last accessed on 6 Feb 2019)
9. Barnes DE, Yaffe K. The projected effect of risk factor reduction on Alzheimer's disease prevalence. *Lancet Neurol* 2011;10:819-28.
10. Norton S, Matthews FE, Barnes DE, et al. Potential for primary prevention of Alzheimer's disease: an analysis of population-based data. *Lancet Neurol* 2014;13:788-94.
11. Livingston G, Sommerlad A, Orgeta V, et al. Dementia prevention, intervention, and care. *Lancet* 2017;390:2673-734.
12. Sochocka M, Zwolinska K, Leszek J. The Infectious Etiology of Alzheimer's Disease. *Curr Neuropharmacol* 2017;15:996-1009.
13. Lim SL, Rodriguez-Ortiz CJ, Kitazawa M. Infection, systemic inflammation, and Alzheimer's disease. *Microbes Infect* 2015;17:549-56.
14. Mawanda F, Wallace R. Can Infections Cause Alzheimer's Disease? *Epidemiol Rev* 2013;35:161-80.
15. Maheshwari P, Eslick GD. Bacterial infection and Alzheimer's disease: a meta-analysis. *J Alzheimers Dis* 2015;43:957-66.
16. van Gool WA, van de Beek D, Eikelenboom P. Systemic infection and delirium: when cytokines and acetylcholine collide. *Lancet* 2010;375:773-5.
17. Girard TD, Jackson JC, Pandharipande PP, et al. Delirium as a predictor of long-term cognitive impairment in survivors of critical illness. *Crit Care Med* 2010;38:1513-20.
18. MacLullich AM, Beaglehole A, Hall RJ, et al. Delirium and long-term cognitive impairment. *Int Rev Psychiatry* 2009;21:30-42.
19. Davis DH, Muniz Terrera G, Keage H, et al. Delirium is a strong risk factor for dementia in the oldest-old: a population-based cohort study. *Brain* 2012;135:2809-16.
20. Foley NC, Affoo RH, Martin RE. A Systematic Review and Meta-Analysis Examining Pneumonia-Associated Mortality in Dementia. *Dement Geriatr Cogn Disord* 2015;39:52-67.

For peer review only - http://bmjopen.bmj.com/site/about/guidelines.xhtml

21. Chae JH, Miller BJ. Beyond Urinary Tract Infections (UTIs) and Delirium: A Systematic Review of UTIs and Neuropsychiatric Disorders. *J Psychiatr Pract* 2015;21:402-11.
22. Fulop T, Witkowski JM, Bourgade K, et al. Can an Infection Hypothesis Explain the Beta Amyloid Hypothesis of Alzheimer's Disease? *Front Aging Neurosci* 2018;10:224.
23. Itzhaki RF, Lathe R, Balin BJ, et al. Microbes and Alzheimer's Disease. *Journal of Alzheimer's disease : JAD* 2016;51:979-84.
24. Moher D, Shamseer L, Clarke M, et al. Preferred reporting items for systematic review and meta-analysis protocols (PRISMA-P) 2015 statement. *Syst Rev* 2015;4:1.
25. Booth A, Clarke M, Dooley G, et al. The nuts and bolts of PROSPERO: an international prospective register of systematic reviews. *Syst Rev* 2012;1:2.
26. Moher D, Liberati A, Tetzlaff J, et al. Preferred reporting items for systematic reviews and meta-analyses: the PRISMA statement. *PLoS Med* 2009;6:e10000097.
27. Stroup DF, Berlin JA, Morton SC, et al. Meta-analysis of observational studies in epidemiology: a proposal for reporting. Meta-analysis Of Observational Studies in Epidemiology (MOOSE) group. *Jama* 2000;283:2008-12.
28. McGowan J, Sampson M, Salzwedel DM, et al. PRESS Peer Review of Electronic Search Strategies: 2015 Guideline Statement. *J Clin Epidemiol* 2016;75:40-6.
29. Grahl JJ, Stollings JL, Rakhit S, et al. Antimicrobial exposure and the risk of delirium in critically ill patients. *Crit care* 2018;22:337.
30. Matthews F, Brayne C, Medical Research Council Cognitive F, et al. The incidence of dementia in England and Wales: findings from the five identical sites of the MRC CFA Study. *PLoS Med* 2005;2:e193-e.
31. Andersen K, Launer LJ, Dewey ME, et al. Gender differences in the incidence of AD and vascular dementia: The EURODEM Studies. EURODEM Incidence Research Group. *Neurology* 1999;53:1992-7.
32. Karp A, Kareholt I, Qiu C, et al. Relation of education and occupation-based socioeconomic status to incident Alzheimer's disease. *Am J Epidemiol* 2004;159:175-83.
33. Tang MX, Cross P, Andrews H, et al. Incidence of AD in African-Americans, Caribbean Hispanics, and Caucasians in northern Manhattan. *Neurology* 2001;56:49-56.
34. Zhong G, Wang Y, Zhang Y, et al. Smoking is associated with an increased risk of dementia: a meta-analysis of prospective cohort studies with investigation of potential effect modifiers. *PloS One* 2015;10:e0118333-e.
35. Sabia S, Fayosse A, Dumurgier J, et al. Alcohol consumption and risk of dementia: 23 year follow-up of Whitehall II cohort study. *BMJ* 2018;362:k2927.
36. Justin BN, Turek M, Hakim AM. Heart disease as a risk factor for dementia. *Clin Epidemiol* 2013;5:135-45.
37. Gudala K, Bansal D, Schifano F, et al. Diabetes mellitus and risk of dementia: A meta-analysis of prospective observational studies. *J Diabetes Investig* 2013;4:640-50.
38. Deckers K, Camerino I, van Boxtel MPJ, et al. Dementia risk in renal dysfunction: A systematic review and meta-analysis of prospective studies. *Neurology* 2017;88:198-208.
39. Zilkens RR, Bruce DG, Duke J, et al. Severe psychiatric disorders in mid-life and risk of dementia in late- life (age 65-84 years): a population based case-control study. *Curr Alzheimer Res* 2014;11:681-93.
40. Kuźma E, Lourida I, Moore SF, et al. Stroke and dementia risk: A systematic review and meta-analysis. *Alzheimers Dement* 2018;14:1416-26.
41. Dodd JW. Lung disease as a determinant of cognitive decline and dementia. *Alzheimers Res Ther* 2015;7:32.

For peer review only - http://bmjopen.bmj.com/site/about/guidelines.xhtml

42. Bhaskaran K, Mussini C, Antinori A, et al. Changes in the incidence and predictors of human immunodeficiency virus–associated dementia in the era of highly active antiretroviral therapy. *Ann Neurol* 2008;63:213-21.
43. Sterne JA, Hernán MA, Reeves BC, et al. ROBINS-I: a tool for assessing risk of bias in non-randomised studies of interventions. *BMJ* 2016;355.
44. Higgins JPT, Altman DG, Gøtzsche PC, et al. The Cochrane Collaboration's tool for assessing risk of bias in randomised trials. *BMJ* 2011;343.
45. Riley RD, Higgins JPT, Deeks JJ. Interpretation of random effects meta-analyses. *BMJ* 2011;342.
46. Higgins J, Green S. Section 9.5.2. Identifying and measuring heterogeneity in Cochrane handbook for systematic reviews of interventions 2011 [Internet] https://handbook-5-1.cochrane.org/chapter_9/9_5_2_identifying_and_measuring_heterogeneity.htm; (Last accessed on 8 Mar 2019)
47. Sterne JA, Egger M. Funnel plots for detecting bias in meta-analysis: guidelines on choice of axis. *J Clin Epidemiol* 2001;54:1046-55.
48. Guyatt GH, Oxman AD, Vist GE, et al. GRADE: an emerging consensus on rating quality of evidence and strength of recommendations. *BMJ* 2008;336:924-6.
49. Balshem H, Helfand M, Schünemann HJ, et al. GRADE guidelines: 3. Rating the quality of evidence. *J Clin Epidemiol* 2011;64:401-6.

For peer review only

For peer review only - http://bmjopen.bmj.com/site/about/guidelines.xhtml

Appendix 1. Medline (OVID) search strategy

1. Pneumonia/ or pneumonia, bacterial/
2. Pneumonia.ti,ab
3. Lower respiratory tract infection*.ti,ab
4. (LRTI or LRTIS).ti,ab.
5. Exp urinary tract infections/
6. (Urinary adj5 infection*).ti,ab.
7. (UTI or UTIS).ti,ab
8. exp Cystitis/
9. (bacteriuria or pyuria or cystitis or pyelonephritis or cellulitis).ti,ab.
10. exp cellulitis/
11. (Skin and soft tissue infection).mp.
12. Exp sepsis/
13. (septic* or sepsis or septic?emia or systemic inflammatory response syndrome or blood stream infection or py?emia).ti,ab.
14. 1 or 2 or 3 or 4 or 5 or 6 or 7 or 8 or 9 or 10 or 11 or 12 or 13
15. Exp dementia/
16. Exp prion diseases/
17. (huntington* or kluver-bucy or prion disease or Creutzfeldt-jakob or primary progressive aphasia).ti,ab
18. (Dement* or Alzheimer*).ti,ab
19. (Lewy*adj2 bod*).ti,ab.
20. Cognitive dysfunction/
21. (Mild cognitive impairment or MCI).ti,ab
22. ((cognit* or memory or cerebr* or mental*) adj3 (declin* or impair* or los* or deteriorat* or degenerat* or complain* or disturb* or disorder*)).ti,ab.
23. Cognitive function.ti,ab.
24. 15 or 16 or 17 or 18 or 19 or 20 or 21 or 22 or 23
25. cohort studies/ or longitudinal study/ or follow-up study/ or prospective study/ or retrospective study/ or cohort.ti,ab. or longitudinal.ti,ab. or prospective.ti,ab. or retrospective.ti,ab.
26. Case-Control Studies/ or Control Groups/ or Matched-Pair Analysis/ or ((case* adj5 control*) or (case adj3 comparison*) or control group*).ti,ab.
27. Incidence/ or incidence.ti,ab,kw.
28. (hazard ratio or HR or odds ratio or relative risk or RR).ti,ab.
29. 25 or 26 or 27 or 28
30. 14 and 24 and 29

For peer review only

Reporting checklist for protocol of a systematic review.

Based on the PRISMA-P guidelines.

Instructions to authors

Complete this checklist by entering the page numbers from your manuscript where readers will find each of the items listed below.

Your article may not currently address all the items on the checklist. Please modify your text to include the missing information. If you are certain that an item does not apply, please write "n/a" and provide a short explanation.

Upload your completed checklist as an extra file when you submit to a journal.

In your methods section, say that you used the PRISMA-Preporting guidelines, and cite them as:

Moher D, Shamseer L, Clarke M, Ghersi D, Liberati A, Petticrew M, Shekelle P, Stewart LA. Preferred Reporting Items for Systematic Review and Meta-Analysis Protocols (PRISMA-P) 2015 statement. Syst Rev. 2015;4(1):1.

		Reporting Item	Page Number
**Title**			
Identification	#1a	Identify the report as a protocol of a systematic review	1
Update	#1b	If the protocol is for an update of a previous systematic review, identify as such	n/a
**Registration**			
	#2	If registered, provide the name of the registry (such as PROSPERO) and registration number	2
**Authors**			
Contact	#3a	Provide name, institutional affiliation, e-mail address of all protocol authors; provide physical mailing address of corresponding author	1
Contribution	#3b	Describe contributions of protocol authors and identify the guarantor of the review	9
**Amendments**			

For peer review only

	#4	If the protocol represents an amendment of a previously completed or published protocol, identify as such and list changes; otherwise, state plan for documenting important protocol amendments	n/a
**Support**			
Sources	#5a	Indicate sources of financial or other support for the review	9
Sponsor	#5b	Provide name for the review funder and / or sponsor	9
Role of sponsor or funder	#5c	Describe roles of funder(s), sponsor(s), and / or institution(s), if any, in developing the protocol	9
**Introduction**			
Rationale	#6	Describe the rationale for the review in the context of what is already known	4/5
Objectives	#7	Provide an explicit statement of the question(s) the review will address with reference to participants, interventions, comparators, and outcomes (PICO)	5
**Methods**			
Eligibility criteria	#8	Specify the study characteristics (such as PICO, study design, setting, time frame) and report characteristics (such as years considered, language, publication status) to be used as criteria for eligibility for the review	5/6
Information sources	#9	Describe all intended information sources (such as electronic databases, contact with study authors, trial registers or other grey literature sources) with planned dates of coverage	6/7
Search strategy	#10	Present draft of search strategy to be used for at least one electronic database, including planned limits, such that it could be repeated	7 (and in appendices))
Study records - data management	#11a	Describe the mechanism(s) that will be used to manage records and data throughout the review	7
Study records - selection process	#11b	State the process that will be used for selecting studies (such as two independent reviewers) through each phase of the review (that is, screening, eligibility and inclusion in meta-analysis)	7

For peer review only

Study records - data collection process	#11c	Describe planned method of extracting data from reports (such as piloting forms, done independently, in duplicate), any processes for obtaining and confirming data from investigators	7
Data items	#12	List and define all variables for which data will be sought (such as PICO items, funding sources), any pre-planned data assumptions and simplifications	7/8
Outcomes and prioritization	#13	List and define all outcomes for which data will be sought, including prioritization of main and additional outcomes, with rationale	6
Risk of bias in individual studies	#14	Describe anticipated methods for assessing risk of bias of individual studies, including whether this will be done at the outcome or study level, or both; state how this information will be used in data synthesis	8
Data synthesis	#15a	Describe criteria under which study data will be quantitatively synthesised	8
Data synthesis	#15b	If data are appropriate for quantitative synthesis, describe planned summary measures, methods of handling data and methods of combining data from studies, including any planned exploration of consistency (such as I2, Kendall's τ)	8
Data synthesis	#15c	Describe any proposed additional analyses (such as sensitivity or subgroup analyses, meta-regression)	8
Data synthesis	#15d	If quantitative synthesis is not appropriate, describe the type of summary planned	8
Meta-bias(es)	#16	Specify any planned assessment of meta-bias(es) (such as publication bias across studies, selective reporting within studies)	9
Confidence in cumulative evidence	#17	Describe how the strength of the body of evidence will be assessed (such as GRADE)	9

Notes:

- 10: 7 (and in appendices)) The PRISMA-P checklist is distributed under the terms of the Creative Commons Attribution License CC-BY 4.0. This checklist was completed on 09. June 2019 using https://www.goodreports.org/, a tool made by the EQUATOR Network in collaboration with Penelope.ai

For peer review only
